# Airway clearance technique therapy for atelectasis induced by scoliosis surgery: a case report

**DOI:** 10.3389/fmed.2025.1518935

**Published:** 2025-02-11

**Authors:** Rui Zhai, Hairong Su, Yaxu Wu, Rong Tan, Xiaoli Zhang, Ye Tian, Mei Hu

**Affiliations:** ^1^Department of Critical Care Medicine, Ninth Medical Center, General Hospital of the People’s Liberation Army, Beijing, China; ^2^Department of Spinal Surgery, Ninth Medical Center, General Hospital of the People’s Liberation Army, Beijing, China

**Keywords:** airway clearance technique, severe scoliosis, atelectasis, postoperative complications of scoliosis, case report

## Abstract

The patient, a 55-year-old female presenting with spinal deformity and exertional dyspnea, was referred to the hospital. Radiographic evaluation of her spine revealed an “S”-shaped scoliosis with a Cobb angle measuring 68°, indicative of severe scoliosis. Despite receiving medication for expectoration, postoperative symptoms including chest tightness, breathlessness, and ineffective coughing persisted and progressively worsened. Subsequent chest CT scans demonstrated extensive atelectasis, and pharmacological interventions proved to be ineffective. Considering the patient’s clinical condition, we implemented airway clearance technique (ACT) along with prone ventilation to optimize cough effectiveness and mitigate atelectasis formation. The airway clearance techniques (ACT) employed include nebulization, continuous positive expiratory pressure (CPEP), and continuous high frequency oscillation (CHFO). Chest CT imaging confirmed that ACT substantially alleviated the patient’s pulmonary atelectasis. Moreover, blood gas analysis indicated significant improvements in both the PaO_2_/FiO_2_ ratio and base excess of whole blood. Follow-up evaluation 1 year post-discharge revealed a favorable prognosis for the patient. We anticipate that our experience utilizing these novel therapeutic modalities will provide valuable insights for clinicians managing similar complications.

## Introduction

The primary diagnostic criterion for scoliosis is a spinal curvature exceeding 10° on anterior and posterior X-rays. In the absence of other abnormal symptoms, such as hemivertebrae, it is referred to as idiopathic scoliosis (1). Despite the unknown pathogenesis of scoliosis, a review suggests four main pathogenetic mechanisms based on reported evidence: asymmetric bone growth dysregulation, susceptibility of bones to deformation, abnormal passive or disturbed active spinal system maintenance (2). Scoliosis restricts rib movement and impairs respiratory muscle function while displacing thoracic organs. Severe cases may lead to respiratory failure (3). Adult patients commonly experience pain and neurological symptoms compared to adolescent patients, with more complex procedures associated with higher rates of intraoperative and perioperative complications (4). A meta-analysis, which synthesized data from 17 clinical trials, demonstrated that the overall incidence of postoperative complications following scoliosis surgery was 23%, with pulmonary complications affecting 6.7% of cases (5). This emphasizes the significance of therapeutic management for postoperative complications. Herein we present a case involving severe pulmonary atelectasis as a postoperative complication following scoliosis surgery. When conventional treatment regimens proved ineffective, our innovative approach utilizing noninvasive airway clearance technique combined with existing research led to significant improvements in patients suffering from atelectasis. We aim to provide clinicians with novel insights into managing such challenges.

Airway clearance techniques (ACT) are non-invasive interventions aimed at enhancing sputum clearance, thereby optimizing ventilation and mitigating the effects of coughing and dyspnea (6). ACT typically consists of four essential components. First, nebulization is utilized to loosen sputum or administer therapeutic medications. Second, continuous positive expiratory pressure (CPEP) is applied to maintain airway patency and dilation. Third, continuous high frequency oscillation (CHFO), a type of pneumatic chest physiotherapy, employs continuous high frequency oscillation within the airways to facilitate sputum mobilization. Finally, patients actively participate in expectoration. ACT is currently employed in the management of bronchiectasis, chronic suppurative lung disease, and cystic fibrosis (6–8). Pulmonary atelectasis is commonly managed through infection control, oxygen therapy, respiratory support, and surgical intervention when necessary. In this specific case, however, the patient exhibited inadequate response to conventional treatments including oxygen therapy, ambroxol hydrochloride, and budesonide. Given the patient’s refusal of invasive procedures, the medical team faced the challenge of effectively managing the atelectasis while simultaneously alleviating the patient’s discomfort. To address this issue, the physicians conducted an extensive literature review to learn the background and efficacy of airway clearance techniques (ACT). Based on clinical experience indicating ACT’s potential in treating post-scoliosis surgery atelectasis, they discussed the treatment options with the patient and ultimately decided to proceed with ACT therapy. This innovative application of ACT not only successfully resolved the atelectasis but also minimized pain associated with invasive procedures and reduced overall treatment costs.

## Case report

The patient, a 55-year-old female, had a surgical history of hydrocephalus correction at 6 weeks of age and underwent right-sided hip replacement 43 years ago. Additionally, she had a medical history of COPD. Following birth, the patient presented with spinal deformity characterized by deviation to one side. The patient eventually sought medical attention for curvature of the spine, chest tightness, and breath-holding with activity and continued aggravation.

**Figure 1:**
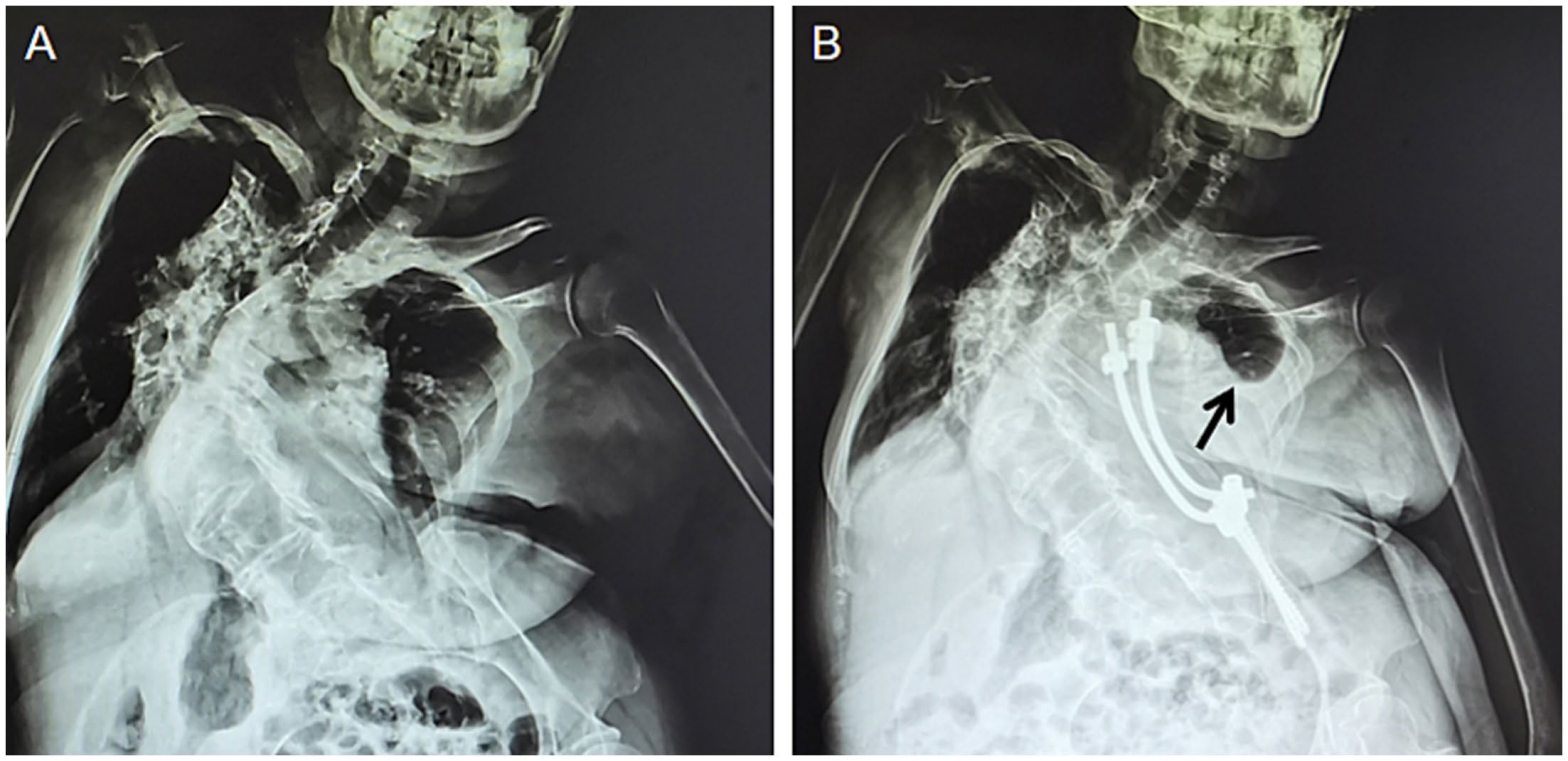
Radiological images of the patient’s spine. **(A)** The preoperative state. **(B)** The postoperative state. The black arrow points to the location of pulmonary atelectasis in the patient’s left lung.

**Figure 2:**
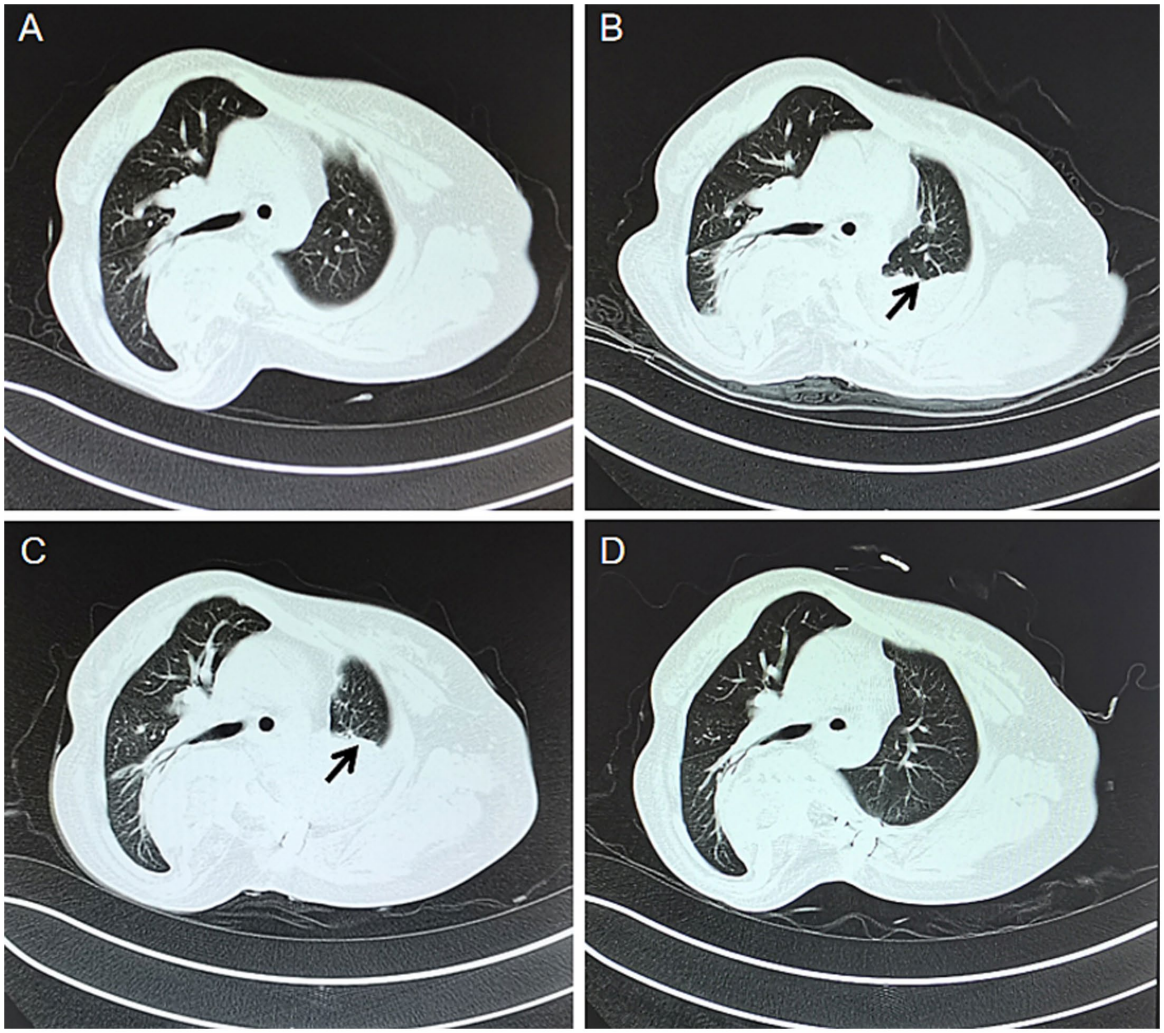
CT images of the patient’s chest at different times. **(A)** Preoperative. **(B)** Post-surgery at 2 days. **(C)** Post-surgery at 13 days. **(D)** One week after airway clearance treatment. The black arrow points to the location of pulmonary atelectasis in the patient’s left lung.

**Figure 3:**
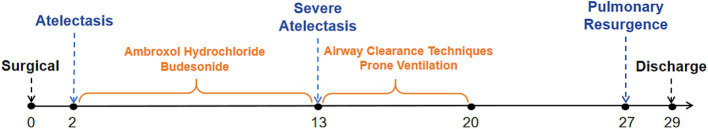
Roadmap of the time when the patient develops a post-operative complication—atelectasis and its treatment.

On physical examination, the patient’s height was 150 cm and weight was 42 kg. The body temperature was 36.1°C, pulse rate was 78 beats per minute, respiratory rate was 18 breaths per minute, and blood pressure was 110/70 mmHg. On thoracic auscultation, bilateral lung fields were clear; however, breath sounds were diminished. Following physical examination, it was determined that the patient exhibited a 4 cm elevation of the right shoulder compared to the left shoulder, a 3 cm elevation of the left side of the pelvis compared to the right side, unequal lower limb lengths, and distances from the anterior superior iliac crest to inner ankle measuring 77 cm on the left side and 74 cm on the right side. No abnormalities were observed in other areas of the patient’s body. Ancillary X-rays (Figure 1A) and CT scans (Figure 2A) revealed an “S”-shaped scoliosis with a right convex kyphoscoliosis exhibiting a Cobb angle of 68° along with some vertebral dysplasia; the first 7, 9, and 10 thoracic vertebrae exhibited hemivertebrae with spinal cord cavities in cervical segments 4–6, longitudinal fissures of the spinal cord at the cervical-lumbar level, and formation of bony ridges in the spinal canal at the thoracic-lumbar level; bilateral lung striations with localised incomplete expansion of lung tissue. The results of blood gas analyses are summarized in Table 1. Detailed outcomes of pulmonary function tests are provided in Table 2; however, it should be noted that several tests were incompletely performed due to the patient’s limited lung vital capacity. Given these findings, particularly the presence of hemivertebrae, the preliminary diagnosis was scoliosis with associated pulmonary dysfunction. Successful posterior orthopedic implant fusion and internal fixation for scoliosis were achieved under general anesthesia using a standardized anesthetic regimen, which included sufentanil 50 μg, cisatracurium besylate 10 mg, midazolam 10 mg, etomidate 20 mg, remifentanil 2 mg, and propofol 1 g. The operation lasted 145 min. To enhance the clarity of the treatment process, we have formulated a detailed timeline as illustrated in Figure 3. The patient reported a VAS pain score of 3 on the first postoperative day, which may have inadvertently contributed to a reduced frequency of coughing. This reduction in coughing frequency could have subsequently led to symptoms of chest tightness, dyspnea, and difficulty expectorating that evening, persisting into the second postoperative day. The VAS pain rating scale is provided in Supplementary Figure S1. A chest CT examination conducted on the second day after surgery revealed bilateral lung striations, localized lung tissue distension insufficiency that was more progressive than preoperative findings, bilateral pleural effusions, and atelectasis (Figures 2A,B). The patient’s VAS pain score decreased to 0 by the fourth post-operative day. Initial treatment with ambroxol hydrochloride injection (4 mL) and nebulized inhalation of budesonide suspension (1 mg) proved ineffective as symptoms worsened progressively. Subsequent chest CT at 13 days after operation indicated further exacerbation of atelectasis (Figure 2C), while radiological examination confirmed postoperative scoliosis (Figure 1B). Blood gas analysis revealed significant deterioration in several critical parameters, including the PaO_2_/FiO_2_ ratio, base excess in whole blood, and actual bicarbonate concentration (Table 1). The patient would not accept invasive treatment. Based on the original programme, an individualized airway clearance technique (ACT) protocol was developed considering the patient’s specific condition. The ACT programme involved nebulisation with sterilised water for injection (10 mL) for 5 min, followed by continuous positive expiratory pressure (CPEP) for 2.5 min to promote lung expansion, and then switching to continuous high frequency oscillation (CHFO) for 2.5 min to facilitate sputum clearance, the above procedure is repeated a total of three times. ACT takes place once a day at 8/12/16 pm for 7 days. The treatment was administered in the prone position for a duration of 8 h per day along with respiratory muscle exercises, while the patient received instructions on effective coughing techniques. No adverse effects were observed during the course of treatment, which resulted in significant alleviation of the patient’s symptoms that did not recur within 1 week after completion. Subsequent chest CT scans revealed significant improvement in pulmonary atelectasis (Figure 2D). Blood gas analysis also demonstrated substantial improvements in PaCO_2_ levels, the PaO_2_/FiO_2_ ratio, whole blood base excess, and actual bicarbonate concentrations (Table 1). In light of the patient’s suboptimal spirometry results, pulmonary function tests were not performed during the course of treatment. Prior to discharge, we reassessed the patient’s pulmonary function and noted that although certain parameters, including FEV1/FVC, had demonstrated improvement, overall lung function remained less than optimal, providing only partial data (Table 2). The patient was discharged 9 days after the completion of ACT treatment. Discharge advice included avoiding strenuous exercise for 3 months, maintaining moderate low back muscle function exercise, adhering to respiratory function exercises, and scheduling regular follow-up appointments. The patient reported an excellent recovery and exhibited no abnormal symptoms during the 1-year follow-up after discharge. The patient expressed high satisfaction with the treatment outcomes.

**Table 1:** Blood gas analysis results of patient.

Variable	Reference range, this hospital^a^	Admission	Day of surgery	1 day after surgery	13 days after surgery	Final test
Partial pressure of arterial oxygen (PaO_2_), mmHg	83–108	71	103	130	85	68
Partial pressure of arterial carbon dioxide (PaCO_2_), mmHg	35–45	57	67	62	62	45
PH	7.35–7.45	7.40	7.18	7.35	7.41	7.38
PaO_2_/FiO_2_ ratio, mmHg	>300	338	490	510	258	324
Base excess of whole blood (BEb), mmol/L	−3 to +3	8.8	−3.8	7.1	12.4	1
Actual bicarbonate (AB), mmol/L	22–26	35.3	25	34.2	39.3	26.6

a Reference values are influenced by many variables, including the patient population and the laboratory methods used. Therefore, our reference values may not apply to all patients.

**Table 2:** Patient pulmonary function test results.

Variable	Predicted values	Admission	Discharge
VC MAX, L	2.39	0.68	0.59
FVC, L	2.33	0.64	0.57
FEV1, L	1.95	0.4	0.4
FEV1/FVC, %	83.69	61.94	69.92
MVV, L/min	83.9	12.76	13.89
R5, cmH_2_O/(L/s)	3.94	15.33	7.69
R20, cmH_2_O/(L/s)	3.32	7.99	5.08
X5, cmH_2_O/(L/s)	−0.71	−2.12	−4.06

VC, vital capacity; FVC, forced vital capacity; FEV1, forced expiratory volume in 1 second; MVV, maximum voluntary ventilation; R5, resistance at 5 Hz; R20, resistance at 20 Hz; X5, reactance at 5 Hz.

## Discussion

Different surgical approaches for scoliosis treatment can impact pulmonary function and increase the risk of postoperative pulmonary complications (9). In adults with scoliosis, surgical interventions often present complex scenarios (10). Furthermore, a study demonstrated that approximately 2.3% of patients undergoing surgery for congenital scoliosis experienced postoperative complications related to atelectasis (11). In this particular case, the patient exhibited pre-existing pulmonary dysfunction and a history of COPD, which further complicated the procedure and escalated the risk of complications (12). Although the literature on postoperative complications of scoliosis has been documented, no relevant reports specifically address strategies for severe cases of atelectasis.

Atelectasis is a frequently encountered mechanical complication during the perioperative period, characterized by inadequate lung expansion into the chest wall (13, 14). Atelectasis not only impairs oxygenation and reduces lung compliance but also triggers inflammation, damages the alveolar-capillary barrier, and may lead to severe lung injury (14–16). Therefore, effective management of atelectasis is crucial for ensuring patient health and safety. Although continuous positive airway pressure ventilation has been reported as a preventive measure against partial lung collapse during anesthesia induction (17), it has not been considered as the treatment of choice for atelectasis (18). With the innovative application of ACT therapy tailored to individual patient’s specific conditions, we achieved a more satisfactory therapeutic outcome in managing postoperative complications associated with atelectasis.

ACT is a non-invasive therapeutic modality that primarily utilizes continuous positive expiratory pressure (CPEP) ventilation to maintain airway patency, enhance alveolar ventilation, and promote sputum expulsion, thereby achieving its therapeutic objectives continuous high frequency oscillation. Additionally, it employs continuous high frequency oscillation to loosen secretions, while simultaneously accelerating ciliary movement to facilitate the migration of peripheral bronchial secretions towards the larger airways. In cases where pharmacological interventions are ineffective, clinicians actively explore alternative treatments to alleviate patient suffering. After conducting a comprehensive review of available literature to fully understand the benefits and limitations of ACT, and considering this information alongside their clinical experience, the clinician ultimately selected ACT as the treatment modality. The procedure and timing were also chosen in accordance with the European Respiratory Society’s guidelines on airway clearance techniques for adults with bronchiectasis (19). To facilitate the patient’s recovery, we implemented a prone position protocol based on clinical evidence from studies of COVID-19 patients, providing 8 h of daily prone positioning (20, 21). The patient’s left lung was successfully re-expanded and blood gas indices were significantly improved through the implementation of ACT therapy (Figure 2; Table 1).

In this instance, ACT treatment played a crucial role in reversing atelectasis, a complication that can occur following lateral spinal surgery. This highlights the significant potential of ACT for managing postoperative pulmonary atelectasis associated with similar surgical procedures. The notable efficacy of ACT is consistent with the well-documented effectiveness of radiofrequency oscillation in addressing atelectasis in perioperative contexts (22). When considering the application of ACT, it is crucial for physicians to possess a thorough understanding of its mechanism of action and the specific advantages and limitations of each technique, as outlined in authoritative guidelines such as the European Respiratory Society statement, in order to mitigate potential unforeseen complications (19). However, individual cases exhibit unique characteristics, and it remains uncertain whether ACT is universally applicable to all patients who develop post-operative atelectasis. Future research should prioritize the design and implementation of rigorous randomized controlled clinical trials to ascertain the efficacy of ACT in addressing this particular postoperative complication.

## Data Availability

The original contributions presented in the study are included in the article/Supplementary material, further inquiries can be directed to the corresponding author.

## Supplementary material

SUPPLEMENTARY FIGURE S1 VAS pain rating scale. The pain levels in the table are in ascending order from 0 to 10, where 0 is no pain and 10 is severe pain. The patient ticks the appropriate number for his/her situation.

## References

[1] Trobisch P Suess O Schwab F. Idiopathic scoliosis. Dtsch Arztebl Int. (2010) 107:875–84. doi: 10.3238/arztebl.2010.0875, PMID: 21191550 PMC3011182

[2] De Sèze M Cugy E. Pathogenesis of idiopathic scoliosis: a review. Ann Phys Rehabil Med. (2012) 55:128–38. doi: 10.1016/j.rehab.2012.01.003, PMID: 22321868

[3] Koumbourlis AC. Scoliosis and the respiratory system. Paediatr Respir Rev. (2006) 7:152–60. doi: 10.1016/j.prrv.2006.04.009, PMID: 16765303

[4] Heary RF. Evaluation and treatment of adult spinal deformity. Invited submission from the Joint Section Meeting on Disorders of the Spine and Peripheral Nerves, March 2004. J Neurosurg Spine. (2004) 1:9–18. doi: 10.3171/spi.2004.1.1.0009 15291014

[5] Roser MJ Askin GN Labrom RD Zahir SF Izatt M Little JP. Vertebral body tethering for idiopathic scoliosis: a systematic review and meta-analysis. Spine Deform. (2023) 11:1297–307. doi: 10.1007/s43390-023-00723-9, PMID: 37432604 PMC10587225

[6] O’Neill K O’Donnell AE Bradley JM. Airway clearance, mucoactive therapies and pulmonary rehabilitation in bronchiectasis. Respirology. (2019) 24:227–37. doi: 10.1111/resp.13459, PMID: 30650472

[7] Schofield LM Singh SJ Yousaf Z Wild JM Hind D. Personalising airway clearance in chronic suppurative lung diseases: a scoping review. ERJ Open Res. (2023) 9:00010-2023. doi: 10.1183/23120541.00010-2023 37342087 PMC10277870

[8] Heinz KD Walsh A Southern KW Johnstone Z Regan KH. Exercise versus airway clearance techniques for people with cystic fibrosis. Cochrane Database Syst Rev. (2022) 6:CD013285. doi: 10.1002/14651858.CD013285, PMID: 35731672 PMC9216233

[9] Bullmann V Schulte TL Schmidt C Gosheger G Osada N Liljenqvist UR. Pulmonary function after anterior double thoracotomy approach versus posterior surgery with costectomies in idiopathic thoracic scoliosis. Eur Spine J. (2013) 22:164–71. doi: 10.1007/s00586-012-2316-x, PMID: 22534955 PMC3616475

[10] Aebi M. The adult scoliosis. Eur Spine J. (2005) 14:925–48. doi: 10.1007/s00586-005-1053-9, PMID: 16328223

[11] Yin S Tao H Du H Feng C Yang Y Yang W. Postoperative pulmonary complications following posterior spinal instrumentation and fusion for congenital scoliosis. PLoS One. (2018) 13:e0207657. doi: 10.1371/journal.pone.0207657, PMID: 30444905 PMC6239341

[12] Lagier D Zeng C Fernandez-Bustamante A Vidal Melo MF. Perioperative pulmonary atelectasis: part II. Clinical implications. Anesthesiology. (2022) 136:206–36. doi: 10.1097/ALN.0000000000004009, PMID: 34710217 PMC9885487

[13] Gillett D Mitchell MA Dhaliwal I. Avoid the trap: nonexpanding lung. Chest. (2021) 160:1131–6. doi: 10.1016/j.chest.2021.04.025, PMID: 33895128

[14] Zeng C Lagier D Lee JW Vidal Melo MF. Perioperative pulmonary atelectasis: part I. biology and mechanisms. Anesthesiology. (2022) 136:181–205. doi: 10.1097/ALN.0000000000003943 34499087 PMC9869183

[15] Nguyen DM Mulder DS Shennib H. Altered cellular immune function in the atelectatic lung. Ann Thorac Surg. (1991) 51:76–80. doi: 10.1016/0003-4975(91)90454-x, PMID: 1985580

[16] Duggan M McCaul CL McNamara PJ Engelberts D Ackerley C Kavanagh BP. Atelectasis causes vascular leak and lethal right ventricular failure in uninjured rat lungs. Am J Respir Crit Care Med. (2003) 167:1633–40. doi: 10.1164/rccm.200210-1215OC 12663325

[17] Tusman G Böhm SH. Prevention and reversal of lung collapse during the intra-operative period. Best Pract Res Clin Anaesthesiol. (2010) 24:183–97. doi: 10.1016/j.bpa.2010.02.006, PMID: 20608556

[18] Venus B. CPAP not the treatment of choice for atelectasis. Chest. (1983) 83:586–7. doi: 10.1378/chest.83.3.586b, PMID: 6337790

[19] Herrero-Cortina B Lee AL Oliveira A O'Neill B Jácome C Dal Corso S. European Respiratory Society statement on airway clearance techniques in adults with bronchiectasis. Eur Respir J. (2023) 62:2202053. doi: 10.1183/13993003.02053-2022, PMID: 37142337

[20] Bargoud CG Jih T Baskar D Volk L Siddiqui S Suaray M. Compliance of prone positioning in non-intubated COVID-19 patients. Clin Med Res. (2023) 21:171–6. doi: 10.3121/cmr.2023.1830, PMID: 38296641 PMC11149956

[21] de Araújo MS Santos MMPD de Assis Silva CJ de Menezes RMP Feijão AR de Medeiros SM. Prone positioning as an emerging tool in the care provided to patients infected with COVID-19: a scoping review. Rev Lat Am Enfermagem. (2021) 29:e3397. doi: 10.1590/1518-8345.4732.3397, PMID: 33439949 PMC7798394

[22] Qin YJ Zhang YQ Chen Q Wang Y Li SY. Effect of high-frequency oscillation on reduction of atelectasis in perioperative patients: a prospective randomized controlled study. Ann Med. (2023) 55:2272720. doi: 10.1080/07853890.2023.2272720, PMID: 37874665 PMC10836273

