# Supplementary figures and images for: Airway clearance technique therapy for atelectasis induced by scoliosis surgery: a case report
Source: Front Med (Lausanne). 2025 Feb 11;12:1518935. doi: 10.3389/fmed.2025.1518935 (PMC11850244; doi:10.3389/fmed.2025.1518935)

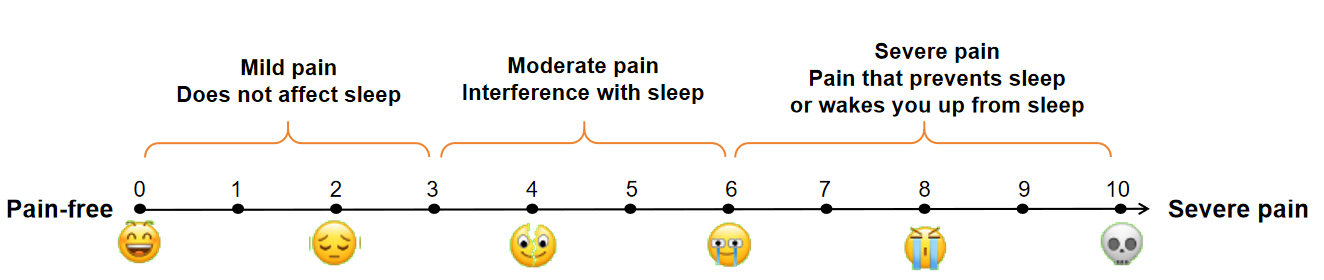

Supplement: SUPPLEMENTARY FIGURE S1 — VAS pain rating scale. The pain levels in the table are in ascending order from 0 to 10, where 0 is no pain and 10 is severe pain. The patient ticks the appropriate number for his/her situation. [file Image_1.jpeg]
